# Polymer-Based Hydrogels Applied in Drug Delivery: An Overview

**DOI:** 10.3390/gels9070523

**Published:** 2023-06-27

**Authors:** Nguyen Hoc Thang, Truong Bach Chien, Dang Xuan Cuong

**Affiliations:** 1 Faculty of Chemical Technology, Ho Chi Minh City University of Food Industry, 140 Le Trong Tan, Tan Phu Distrist, Ho Chi Minh City 700000, Vietnam; chientb@hufi.edu.vn; 2 Innovation and Entrepreneurship Center, Ho Chi Minh City University of Food Industry, 140 Le Trong Tan, Tan Phu Distrist, Ho Chi Minh City 700000, Vietnam; cuongdx@hufi.edu.vn

**Keywords:** hydrogels, drug delivery, multi-sensitive hydrogels, biopolymers, nanoparticles

## Abstract

Polymer-based hydrogels are hydrophilic polymer networks with crosslinks widely applied for drug delivery applications because of their ability to hold large amounts of water and biological fluids and control drug release based on their unique physicochemical properties and biocompatibility. Current trends in the development of hydrogel drug delivery systems involve the release of drugs in response to specific triggers such as pH, temperature, or enzymes for targeted drug delivery and to reduce the potential for systemic toxicity. In addition, developing injectable hydrogel formulations that are easily used and sustain drug release during this extended time is a growing interest. Another emerging trend in hydrogel drug delivery is the synthesis of nano hydrogels and other functional substances for improving targeted drug loading and release efficacy. Following these development trends, advanced hydrogels possessing mechanically improved properties, controlled release rates, and biocompatibility is developing as a focus of the field. More complex drug delivery systems such as multi-drug delivery and combination therapies will be developed based on these advancements. In addition, polymer-based hydrogels are gaining increasing attention in personalized medicine because of their ability to be tailored to a specific patient, for example, drug release rates, drug combinations, target-specific drug delivery, improvement of disease treatment effectiveness, and healthcare cost reduction. Overall, hydrogel application is advancing rapidly, towards more efficient and effective drug delivery systems in the future.

## 1. Introduction

Drug delivery systems (DDS) play a critical role in optimizing the therapeutic efficacy of drugs by addressing the limitations of traditional drug formulations [1,2]. These limitations include low bioavailability, poor solubility, and a short half-life, which can significantly impact drug efficacy and necessitate frequent dosing [2,3]. However, controlled drug delivery systems, such as polymer-based hydrogels [4], offer a promising solution by enabling sustained drug release for a long time [4,5]. This sustained-release property helps maintain therapeutic drug concentrations within the desired range [6], avoiding a sudden increase or decrease that can lead to suboptimal treatment outcomes [7,8,9]. By extending the release duration, polymer-based hydrogels enhance drug bioavailability [8,10] and reduce the frequency of dosing, supporting patient compliance and convenience [7,11]. Additionally, DDSs including polymer-based hydrogels offer the potential for targeted drug delivery to specific tissues or organs [3,8,12]. By incorporating targeting ligands [13] or modifying the polymer-based hydrogel’s properties [10,14], drugs can be directed to their intended sites of action [3,8]. This targeted approach minimizes systemic exposure [15] and reduces the potential for off-target side effects, while maximizing therapeutic efficacy at the desired site [16,17]. Moreover, DDSs can be tailored to accommodate a wide range of drugs with different physicochemical properties [18]. In particular, polymer-based hydrogels offer versatility in drug loading [8] and release mechanisms [19], allowing for the delivery of various types of drugs with small molecules such as proteins [20,21], peptides [22], and nucleic acids [23]. This flexibility makes polymer-based hydrogels an attractive topic for researchers as well as for their applications in the treatment of various medical conditions.

Polymer-based hydrogels are extraordinary materials composed of crosslinked hydrophilic polymers that possess the remarkable ability to absorb and retain large amounts of water and biological fluids while maintaining their three-dimensional structure [24,25]. This unique property, along with their high water content [26,27], porosity [28,29,30,31], and soft consistency [32], makes polymer-based hydrogels highly attractive for a wide range of biomedical applications such as drug delivery [33,34], tissue engineering [35,36], and wound healing [37,38,39]. In fact, polymer-based hydrogels exhibit properties that closely mimic those of natural tissues [40,41], making them an excellent choice for various biomedical applications [17,42,43]. The swelling capability of polymer-based hydrogels is particularly noteworthy, as it allows them to absorb and retain significant amounts of water and biological fluids [44,45], rendering them a highly desirable material for use in medicine and healthcare applications [46,47]. This property enables the polymer-based hydrogels to create a unique environment that facilitates essential biological interactions [48,49], such as cell proliferation [50,51], adhesion [52,53], and differentiation [54,55]. Consequently, polymer-based hydrogels have emerged as promising candidates for tissue engineering [56,57,58] and regenerative medicine applications [59,60].

Polymer-based hydrogels are able to regulate the release of drugs by controlling their swelling behavior leading to sustained drug delivery for a long time [61,62,63]. When loaded with drugs, hydrogels can hold them within their porous structure, gradually releasing them in a controlled manner [63,64]. By modulating the compositions and structures of the hydrogel, the release kinetics and time can be precisely tailored to meet the therapeutic requirements [19,65,66]. This sustained drug release capability of hydrogels offers advantages such as a prolonged therapeutic effect [11,67,68], reduced dosing frequency [3,8,69], and improved patient compliance [8,11]. In addition to sustained drug delivery, polymer-based hydrogels can be engineered to respond to specific stimuli [70,71]. By incorporating responsive components into the hydrogel matrix [72,73], such as temperature-sensitive polymers [74,75] or pH-sensitive moieties [76,77,78], drug release can be triggered [32,79,80] or modulated upon exposure to specific environmental cues [65]. This feature enables targeted drug delivery, where the hydrogel selectively releases the drug at the site of action [81,82], minimizing systemic side effects [8,11,83] and maximizing therapeutic efficacy [6,11,84,85].

The unique properties of polymer-based hydrogels make them versatile for various medical [86,87,88] and healthcare applications [3,8,11,17]. Their biocompatibility, soft consistency, and high water content allow polymer-based hydrogels to closely resemble living tissues [32,41,89], making them suitable for biomedical applications such as tissue engineering [2,56,57,58,90] and regenerative medicine [42,91]. Polymer-based hydrogels can provide a supportive three-dimensional scaffold for cells to proliferate [92,93], adhere, and differentiate, facilitating tissue regeneration [41,89] and repair processes [94,95,96,97]. Moreover, the ability of polymer-based hydrogels to absorb and retain water creates a favorable environment for cellular interactions [98,99,100], promoting favorable biological responses [101,102]. Beyond drug delivery and tissue engineering, hydrogels find utility in biosensing applications [13,15,103]. By incorporating specific bioactive molecules or sensors into the hydrogel network, changes in the surrounding environment [16,17,29], such as the presence of target analytes, can be detected and transduced into measurable signals [81,104,105]. This capability opens up opportunities for the development of diagnostic platforms [11,83,106], implantable biosensors [13,15,69,107,108], and smart drug delivery systems [11,56,109].

The current article presents several notable features and innovations compared to previously published articles in similar fields with analysis and evaluation on the hydrogels produced from polymers applied in drug delivery systems. It adds to the existing body of knowledge by presenting novel concepts, methodologies, and applications that contribute to advancements in the field. These innovations have the potential to enhance the efficacy, safety, and patient compliance of drug delivery systems, paving the way for more modern changes in biomedicine materials.

## 2. Classification of Drug Delivery Systems

Drug delivery systems play a crucial role in optimizing the therapeutic efficacy of drugs by controlling their release [110,111], targeting specific tissues or organs [12,14,81,82], and reducing potential side effects [8,9,11]. In this article, the classification of drug delivery systems based on conventional and novel systems is presented in Table 1. This classification provides a framework for understanding the different approaches and technologies employed in drug delivery research and development. Drug delivery systems can be classified based on several criteria with two main groups including conventional or traditional drug delivery systems and novel drug delivery systems [3,8,11,112,113].

### 2.1. Conventional Drug Delivery Systems

Conventional drug delivery systems, as the name suggests, refer to the established methods and formulations that have been widely used in pharmaceuticals for many years. These systems have undergone extensive research and have a proven track record of safety and efficacy. They include various dosage forms such as tablets, capsules, injections, and creams. Conventional systems primarily focus on delivering drugs to the target site using passive diffusion or basic release mechanisms. While these systems have been successful in many cases, they often have limitations such as low bioavailability, poor solubility, and short half-life, which can decrease their effectiveness and require frequent dosing [3,8,11,112,113]. However, advancements in formulation techniques, such as the use of excipients and optimization of drug release profiles, have led to significant improvements in conventional drug delivery systems. There are several advantages and disadvantages of traditional DDSs as shown in Table 2.

### 2.2. Novel Drug Delivery Systems

New drug delivery systems have emerged as innovative solutions to overcome the limitations of classical drug delivery approaches and enhance the effectiveness as well as safety of therapeutic interventions [2,8]. These systems involve the utilization of specialized carriers or vehicles that can deliver the drug to the desired target site in a controlled and targeted manner [12,114,115]. There are several examples of novel drug delivery systems that have gained significant attention in recent years such as the rate-preprogrammed new drug delivery system [7,8,10]; activation-modulated drug delivery system [8,113,116]; feedback-regulated drug delivery system [3,112,113]; and site-targeting drug delivery system [3,8,112].

#### 2.2.1. Rate-Preprogrammed New Drug Delivery System

This type of DDS is designed to release drugs at a predetermined rate over a specific period. By incorporating various mechanisms such as diffusion [117,118], erosion, or osmosis [74,118], these systems ensure a sustained and controlled release of the drug [119,120], maintaining therapeutic levels in the body for an extended duration [6,11,47,67,68,69]. Rate-preprogrammed systems offer advantages such as reduced dosing frequency, improved patient compliance, and enhanced therapeutic outcomes [84,85].

#### 2.2.2. Activation-Modulated Drug Delivery System

Activation-modulated systems utilize specific triggers or stimuli to initiate drug release at the target site [32,79,80]. The triggers can be external, such as light [121,122,123], heat [124,125], or magnetic fields [126,127,128], or internal [63], including changes in pH [76,77,78,129,130], temperature [131,132], enzyme levels [24,76,132], or redox conditions [11,16,29]. By incorporating responsive materials or incorporating stimuli-responsive components, these systems enable a precise control over drug release, ensuring that the drug is delivered when and where it is needed most [56,70].

#### 2.2.3. Feedback-Regulated Drug Delivery System

This system employs a feedback mechanism to adjust the drug release rate in response to a physiological signal or drug concentrations in the body [91,133,134]. By integrating sensors [103,135] or biomarkers [136,137], these systems can continuously monitor drug levels or the therapeutic response [11,47,84,85] and modulate the drug release accordingly [65,138,139]. The dynamic control mechanism optimizes drug delivery [140,141], minimizes side effects [142,143], and maximizes therapeutic efficacy [144,145,146].

#### 2.2.4. Site-Targeting Drug Delivery System

The site-targeting drug delivery system aims to deliver drugs directly to the desired site or tissue [12,14,115], minimizing off-target effects [3,104,105] and improving treatment outcomes [8,11]. They can utilize various targeting strategies, including ligand–receptor interactions [13], antibody-mediated targeting [81,82], or active targeting using nanocarriers [147,148]. By enhancing drug accumulation at the target site, these systems enhance therapeutic efficacy while reducing systemic toxicity [3,8,149].

These examples represent the exciting advancements in the field of drug delivery systems, where researchers and scientists are actively exploring new approaches to address the challenges associated with conventional drug delivery. By tailoring drug release kinetics, incorporating responsive elements, utilizing feedback mechanisms, and targeting specific sites, these new drug delivery systems offer improved control, precision, and efficiency in drug administration. This aims to optimize drug efficacy, minimize side effects, and improve patient outcomes. Continued research and development in this field holds great promise for revolutionizing the way drugs are delivered, enabling personalized medicine approaches, and ultimately enhancing the overall quality of healthcare.

### 2.3. Carriers for Drug Delivery Systems

Carriers in drug delivery systems are designed to improve the pharmacokinetics and pharmacodynamics of drugs by addressing their limitations and optimizing their delivery. Carriers are designed to transport drugs to specific sites within the body, enhance drug stability [150,151], control drug release [152,153], and improve therapeutic outcomes [84,85]. These carriers can also enhance drug solubility [71,117,118], protect drugs from degradation [154,155], enable targeted delivery [3,8,112], provide sustained release [64,156], and improve drug stability [150,151] and bioavailability [8,11]. Carriers for drug delivery systems can be classified based on various parameters, such as their composition, structure, size, and mechanism of drug release. Some common classifications include liposomes [68,104,143], polymer micelles [63,144,145], microspheres [157,158,159], inorganic nanoparticles [148,160,161], dendrimers [99,142,162], hydrogels [11,12], and others [163,164,165,166,167,168].

Carriers for drug delivery systems possess several important properties such as biocompatibility, stability, drug-loading capacity, targeting ability, and controlled release. Therefore, the carriers for drug delivery systems play important roles in enhancing drug efficacy, improving patient compliance, and reducing side effects.

## 3. Polymer-Based Hydrogels

Polymer-based hydrogels are a type of soft material composed of three-dimensional polymer networks that have the ability to absorb and retain large amounts of water or biological fluids while maintaining their structural integrity [169,170,171]. These hydrophilic polymers are capable of swelling in the presence of water, resulting in a gel-like structure [172,173,174,175]. Crosslinking within the polymer network gives hydrogels their three-dimensional structure and prevents them from dissolving in water [16,23,174]. The polymers used to construct hydrogels can be either natural [176,177] or synthetic [177,178]. Natural polymers commonly used include alginate [179,180], chitosan [181,182], collagen [64], hyaluronic acid [17,23], and gelatin [183,184]. Synthetic polymers such as polyethylene glycol (PEG) [185,186], polyacrylamide (PAAm) [179,187], and poly(N-isopropylacrylamide) (PNIPAAm) [17,23] are also widely employed in hydrogel synthesis. The choice of polymer depends on the desired properties and intended applications of the hydrogel.

Polymer-based hydrogels possess several unique characteristics that make them attractive for various applications. Their high water content and soft consistency resemble living tissues [188], allowing for better integration [82] and minimizing immune responses [88,108,136]. Hydrogels are highly biocompatible, meaning they are compatible with biological systems and do not typically cause significant adverse reactions [135]. Their porosity enables the efficient transport of nutrients, oxygen, and bioactive molecules to cells residing within the hydrogel matrix [51,72,73,189]. These hydrogels can undergo controlled swelling and exhibit tunable mechanical properties, allowing them to mimic the physical and mechanical properties of natural tissues [190,191]. Additionally, hydrogels can be engineered to respond to external stimuli such as changes in temperature [192,193], pH [193,194], or light, making them valuable for applications such as targeted drug delivery or biosensing [13,15,69,103].

**Figure 1:**
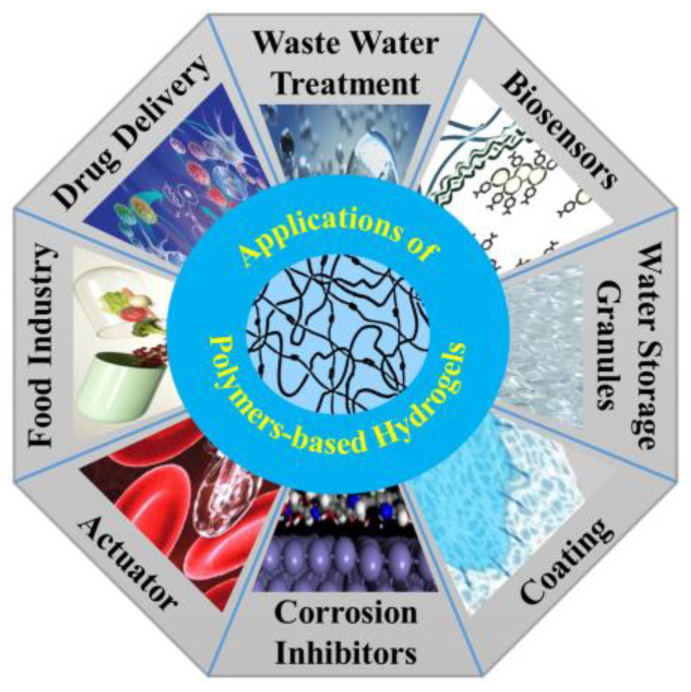
Applications of polymer-based hydrogels for the various engineering fields of the food industry, drug delivery, wastewater treatment, biosensors, water storage granules, coating, corrosion inhibitors, and actuators.

Polymer-based hydrogels have a wide range of applications in various fields, including drug delivery systems [195,196], the food industry [174], wastewater treatment [74], biosensors [103], water storage granules [192,197], coating [108,140], corrosion inhibitors [11,17,23], and actuators [23], as shown in Figure 1. They provide a versatile platform for the controlled release of drugs [111,119], scaffolds for tissue regeneration [198,199], wound dressings [46], and biocompatible materials for medical devices [86]. The design and formulation of polymer-based hydrogels continues to be an active area of research, aiming to develop advanced materials with enhanced properties and functionalities to meet the demands of biomedical applications [9,17,42,43,56,70,87,88,108].

### 3.1. Classifications of Polymer-Based Hydrogels with Their Characteristic Aspects Related to DDSs and Biomedical Applications

There are various classifications of polymer-based hydrogels including based on origin, composition, ionic charge, pore size, physical appearance, configuration, crosslinking, external stimuli response, and others.

#### 3.1.1. Classification of Polymer-Based Hydrogels Based on Origin

Polymer-based hydrogels can be classified based on their origin, which can be natural, synthetic, or a combination of both.

Natural polymer-based hydrogels are hydrogels composed of polymers derived from natural sources, such as plants, animals, or microorganisms. These polymers are biocompatible, biodegradable, and often exhibit inherent bioactivity, making them suitable for a wide range of biomedical applications [154]. Natural polymers include collagen, alginate, chitosan, hyaluronic acid, and gelatin. Natural polymer-based hydrogels offer advantages such as their similarity to the natural extracellular matrix, which supports cell growth and tissue regeneration. They can also provide a favorable microenvironment for encapsulated cells or therapeutic agents [176,177]. Additionally, natural polymers often possess inherent bioactivity, allowing for bioactive molecule incorporation or modification to enhance specific functionalities. The properties and structures of natural polymer-based hydrogels can be modified through various techniques such as crosslinking, blending with other polymers, or incorporating bioactive molecules. This versatility enables the customization of hydrogels to suit specific applications and desired properties [177,178]. Natural polymer-based hydrogels have gained significant attention in the field of regenerative medicine, drug delivery, and biomedical engineering due to their biocompatibility, biodegradability, and inherent bioactivity. Ongoing research and development efforts focus on refining their properties, improving their functionality, and exploring new applications in the fields of tissue engineering, wound healing, and controlled drug release systems [176,177,178].

Synthetic polymer-based hydrogels are hydrogels composed of polymers that are chemically synthesized in the laboratory. These polymers are typically derived from monomers through polymerization reactions, allowing for precise control over their chemical structures, properties, and functionality. Synthetic polymer-based hydrogels offer several advantages, including tunable properties, reproducibility, and the ability to incorporate various functionalities for specific applications [2,18,21,200]. Some common synthetic polymers used in the production of hydrogels are poly(acrylic acid) (PAA) [185,186], poly(N-isopropylacrylamide) (PNIPAAm) [17,23], poly(ethylene glycol) (PEG) [201,202], poly(vinyl alcohol) (PVA) [203,204], poly(HEMA) (hydroxyethyl methacrylate) [81], and others [205,206,207]. Synthetic polymer-based hydrogels offer advantages such as precise control over their chemical and physical properties, mechanical strength, and stability [201,202,203,204,205,206,207]. They can be engineered to exhibit specific characteristics such as controlled drug release [11,17,204], stimuli responsiveness [203], and biodegradability [23,186]. Additionally, synthetic polymers can be modified through various chemical reactions, allowing for the incorporation of bioactive molecules, peptides, or targeting ligands to enhance their functionality and specificity [201,202,203,204,205,206,207]. The properties and structures of synthetic polymer-based hydrogels can be tailored through parameters such as the choice of monomers, polymerization techniques, crosslinking methods, and the introduction of functional groups [204,205,206,207]. This versatility enables the design of hydrogels with specific characteristics suited for different applications. Synthetic polymer-based hydrogels find applications in various fields, including drug delivery systems, tissue engineering, biosensors, and wound healing [11,17,23,201,202,203,204,205,206,207]. Ongoing research focuses on developing novel synthetic polymers, optimizing hydrogel properties, and exploring advanced applications in regenerative medicine, controlled release systems, and biomedical engineering.

Hybrid polymer-based hydrogels, also known as semi-synthetic polymer-based hydrogels, are a type of hydrogel material that combines both natural and synthetic polymers to form a network structure [177,184,208,209]. These hydrogels are created by incorporating natural polymers or biomolecules into a synthetic polymer matrix or by chemically modifying natural polymers with synthetic components [208,209]. The concept of hybrid polymer-based hydrogels stems from the desire to capitalize on the advantages offered by both natural and synthetic polymers. Natural polymers, such as proteins and polysaccharides, provide biocompatibility, bioactivity, and inherent biological functionalities. On the other hand, synthetic polymers offer tunable mechanical properties, control over chemical functionality, and ease of synthesis [11,177]. By combining these two types of polymers, hybrid hydrogels can harness the unique characteristics of each component to achieve the desired properties and functions for specific applications [17,184]. There are various approaches to developing hybrid polymer-based hydrogels known as physical blending and covalent incorporation. For physical blending, natural and synthetic polymers can be physically mixed together to form a hybrid hydrogel [177,208]. The polymers may interact through physical entanglement, hydrogen bonding, or electrostatic interactions. While this method is straightforward, the properties of the resulting hybrid hydrogel are predominantly determined by the individual polymers, and there may be limited chemical integration between the components [23,177,209]. For covalent incorporation, natural polymers can be chemically modified to introduce functional groups that can react with synthetic polymers during the crosslinking process. This covalent incorporation ensures a stronger integration between the natural and synthetic components, leading to a more homogeneous and structurally stable hybrid hydrogel [177,184,209]. Examples of hybrid hydrogels include silk fibroin/PVA hydrogels [210] and chitosan/PEG hydrogels [210]. Hybrid polymer-based hydrogels offer several advantages over pure natural or synthetic polymer hydrogels. They combine the desirable properties of both types of polymers, such as biocompatibility, bioactivity, mechanical strength, and control over properties, to create materials with enhanced performance and functionality [11,17,208,210]. These hydrogels can be tailored to exhibit specific characteristics, such as improved mechanical properties, increased stability, controlled degradation rates, and enhanced drug release profiles [209,210]. The specific properties and structures of hybrid polymer-based hydrogels can be customized by adjusting the composition, ratio, and crosslinking density of the natural and synthetic polymers. This versatility allows for the fine-tuning of properties to suit the requirements of various applications, including tissue engineering, drug delivery, regenerative medicine, and biomedical devices [11,17,23,208,209,210].

#### 3.1.2. Classification of Polymer-Based Hydrogels Based on Composition

Polymer-based hydrogels can also be classified based on their composition, which includes homopolymer [11,17,23,211,212], copolymer [24,30,52,63,67,71,79,98,123], multipolymer [63,213,214], and interpenetrating network (IPN) hydrogels [40,209,215].

Homopolymer-based hydrogels are a type of hydrogel that is composed of a single type of polymer. In other words, the hydrogel network is formed by crosslinking repeating units of the same polymer [11,211]. These hydrogels are created by polymerizing a monomer that consists of identical repeating units, leading to a three-dimensional network structure [17,212]. Homopolymer-based hydrogels have the ability of certain polymers to absorb and retain large amounts of water while maintaining their structural integrity. When the polymer chains are crosslinked, either through chemical or physical interactions, a hydrogel is formed [23,211,212]. The crosslinking allows the polymer chains to hold their positions and prevents them from dissolving or leaching out of the gel structure when exposed to aqueous environments. Homopolymer-based hydrogels can be classified into two main categories of chemically crosslinked homopolymer-based hydrogels and physically crosslinked homopolymer based-hydrogels. In chemically crosslinked homopolymer hydrogels, the crosslinking is achieved through covalent bonds formed between the polymer chains [11,17,212]. This can be accomplished by introducing crosslinking agents during the polymerization process or by post-polymerization crosslinking reactions. Examples of chemically crosslinked homopolymer hydrogels include polyacrylamide (PAAm) hydrogels [179,187], polyethylene glycol (PEG) hydrogels [185,186], and poly(N-isopropylacrylamide) (PNIPAAm) hydrogels [17,23]. For physically crosslinked homopolymer hydrogels, the crosslinking is based on physical interactions, such as entanglements, hydrogen bonding, or hydrophobic interactions between the polymer chains. These interactions are reversible, allowing the hydrogel to swell or shrink in response to external stimuli. Physically crosslinked homopolymer hydrogels are advantageous in terms of their ease of preparation and potential for a stimuli-responsive behavior. Examples of physically crosslinked homopolymer hydrogels include agarose hydrogels, gelatin hydrogels [183,184], and poly(N-vinylcaprolactam) hydrogels [11,17,23]. The properties of homopolymer-based hydrogels can be tailored by adjusting the polymer composition, molecular weight, crosslinking density, and environmental conditions. These hydrogels exhibit characteristics such as a high water absorption capacity, soft and flexible consistency, biocompatibility, and the ability to mimic certain aspects of natural tissues [17,23]. They find applications in various fields, including drug delivery, tissue engineering, wound healing, biosensors, and controlled release systems. While homopolymer-based hydrogels have certain advantages, such as simplicity in their composition and preparation, they may also have limitations [23,211,212]. These can include relatively limited control over the hydrogel properties, lack of specific functionalities, and potential for limited mechanical strength. To address these limitations, researchers often explore the use of copolymers or hybrid systems that incorporate multiple types of polymers or functional groups.

Copolymer-based hydrogels are indeed composed of two or more different monomers that undergo polymerization to form a three-dimensional network [98,123]. These hydrogels offer unique properties that can be tailored based on the combination of monomers used in their synthesis [24,30]. One example of a copolymer-based hydrogel is poly(ethylene glycol)-diacrylate (PEGDA). PEGDA hydrogels are formed by copolymerizing PEGDA monomers with a crosslinking agent such as N,N’-methylenebisacrylamide (BIS). PEGDA hydrogels are biocompatible, exhibit low toxicity, and can be crosslinked under mild conditions. These characteristics make them suitable for drug delivery and tissue engineering applications. Another example is poly(acrylic acid)-co-poly(ethylene glycol) (PAA-co-PEG), which is a copolymer hydrogel composed of two monomers, acrylic acid and ethylene glycol. PAA-co-PEG hydrogels demonstrate pH-sensitive swelling behavior due to the presence of acrylic acid. The acrylic acid units can ionize in basic conditions, leading to the swelling of the hydrogel [11,17,23]. This property makes PAA-co-PEG hydrogels useful for drug delivery applications where a pH-responsive release profile is desired. Copolymer-based hydrogels can be designed to have specific mechanical, swelling, and degradation properties by carefully selecting and adjusting the monomers and their ratios. By controlling these parameters, researchers can tailor the hydrogel’s characteristics to meet the requirements of various applications. The ability to modify copolymer hydrogels makes them versatile materials for fields such as tissue engineering and drug delivery, where precise control over properties is essential. Copolymer-based hydrogels offer the advantage of combining different monomers to achieve unique properties and functionalities [24,30,52,63,67,71,79,98,123]. Examples such as PEGDA and PAA-co-PEG demonstrate their utility in drug delivery systems, tissue engineering, and other applications. The specific combination of monomers determines the properties of hydrogels, enabling researchers to design copolymer hydrogels with the desired characteristics for specific applications.

Multipolymer-based hydrogels are hydrogels composed of three or more different polymer chains. These hydrogels are designed to leverage the beneficial properties of each individual polymer, resulting in a unique combination of properties that can be tailored for specific applications [63,113,114]. There are two common methods for preparing multipolymer hydrogels. The first method involves blending different types of pre-synthesized polymers. This blending process allows for the combination of different polymer chains to achieve the desired properties [63,113]. For example, a blend of chitosan, a natural polymer derived from chitin with excellent biocompatibility and biodegradability, and poly(vinyl alcohol) (PVA), a synthetic polymer with high water solubility and film-forming properties, can create a multipolymer hydrogel with improved mechanical strength, swelling properties, and biocompatibility. The second method for preparing multipolymer hydrogels is through the copolymerization of two or more monomers. For instance, a copolymer of poly(ethylene glycol) (PEG) and poly(lactic acid) (PLA) can be synthesized. PEG, being a hydrophilic polymer, enhances the water uptake of the hydrogel, while PLA, a biodegradable polymer, controls the degradation rate of the hydrogel. By incorporating multiple polymer chains, multipolymer hydrogels offer a broader range of properties compared to single polymer-based hydrogels. The combination of different polymers allows for the fine-tuning of mechanical strength, degradation rates, biocompatibility, and other characteristics [63,113,114]. This versatility makes multipolymer hydrogels suitable for various applications, including tissue engineering, drug delivery, and biomedical implants.

Interpenetrating network (IPN) hydrogels are a type of hydrogel composed of two or more polymer networks that are interlocked at a molecular level. The polymer networks can consist of their homopolymer or copolymer [40,209,215]. The formation of an IPN hydrogel involves the polymerization or crosslinking of the different networks either sequentially or simultaneously. One of the key advantages of IPN hydrogels is their unique combination of properties that arise from the interpenetration of the polymer networks [40,41,42,43,44,45,46,47,48,49,50,51,52,53,54,55,56,57,58,59,60,61,62,63,64,65,66,67,68,69,70,71,72,73,74,75,76,77,78,79,80,81,82,83,84,85,86,87,88,89,90,91,92,93,94,95,96,97,98,99,100,101,102,103,104,105,106,107,108,109,110,111,112,113,114,115,116,117,118,119,120,121,122,123,124,125,126,127,128,129,130,131,132,133,134,135,136,137,138,139,140,141,142,143,144,145,146,147,148,149,150,151,152,153,154,155,156,157,158,159,160,161,162,163,164,165,166,167,168,169,170,171,172,173,174,175,176,177,178,179,180,181,182,183,184,185,186,187,188,189,190,191,192,193,194,195,196,197,198,199,200,201,202,203,204,205,206,207,208,209,215]. By selecting appropriate polymer combinations, the properties of the resulting hydrogel can be tailored to meet specific requirements. For example, the incorporation of different polymers can lead to an improved biocompatibility, mechanical strength, stability, and permeability. The sequential polymerization method involves the formation of one polymer network first, followed by the introduction of the second polymer system, which infiltrates the spaces between the chains of the first network [40,209,215]. In simultaneous polymerization, different monomers or pre-polymers are polymerized together to form the interpenetrating networks. The crosslinking of the polymer networks further strengthens the structure of the IPN hydrogel. IPN hydrogels have a wide range of applications in the biomedical field. In drug delivery systems, IPN hydrogels offer the controlled and sustained release of drugs due to their unique structure and properties. They are also extensively used in tissue engineering, where their enhanced mechanical properties and biocompatibility support cell growth and tissue regeneration [40,209]. IPN hydrogels have shown promise in wound healing applications by providing a moist and favorable environment for tissue repair. Furthermore, their versatility makes them suitable for the development of biosensors that can detect specific analytes or biomarkers. Due to their versatility, IPN hydrogels have a wide range of applications in the biomedical field, including drug delivery, tissue engineering, wound healing, and biosensors [11,17,40,215].

#### 3.1.3. Classification of Polymer-Based Hydrogels Based on Ionic Charge

Polymer-based hydrogels can also be classified based on their ionic charge. These hydrogels can be classified into three types: neutral [129], ionic [216,217], and ampholytic hydrogels [79].

Neutral polymer-based hydrogels are hydrogels that lack an ionic charge. They are composed of polymers that contain hydrophilic groups, such as hydroxyl or amide groups, which enable them to absorb and retain water [11,129]. These neutral polymer-based hydrogels are typically synthesized through the crosslinking polymerization of monomers such as acrylates, methacrylates, or vinyl monomers. These hydrogels offer several advantages in biomedical applications [13,17,129]. Firstly, they are biocompatible and non-toxic, making them suitable for use in contact with biological systems [23,129]. This biocompatibility reduces the risk of adverse reactions or toxicity when the hydrogels are implanted or administered in vivo. Additionally, neutral polymer-based hydrogels can be designed to have specific mechanical properties, such as elasticity or stiffness, which can be tailored to match the requirements of the target tissue or application [11,17,23]. The versatility of neutral hydrogels allows for a wide range of applications in the biomedical field. In drug delivery, they can provide the sustained release of drugs, protecting them from degradation and ensuring their controlled release over time. In tissue engineering, neutral hydrogels can provide a scaffold for cell growth and tissue regeneration, mimicking the natural extracellular matrix [11,17,23]. They can also be used in wound healing to create a moist and protective environment that promotes healing and tissue regeneration.

Ionic polymer-based hydrogels are a type of hydrogel that contain charged functional groups within their polymer structure, such as carboxyl, amine, or sulfonate groups [124,216]. They are responsible for imparting an ionic charge to the hydrogel. When the hydrogel is immersed in water or an aqueous environment these charged groups can dissociate, releasing charged ions into the surrounding solution [174,194,217]. The presence of these charged ions enables interactions with oppositely charged species in the environment. Ionic polymer-based hydrogels can be classified into two main types: cationic polymer-based hydrogels and anionic polymer-based hydrogels, based on the type of charged functional groups they possess [194,216,217]. The cationic polymer-based hydrogels contain positively charged functional groups, typically amine groups. These hydrogels have a propensity to interact with negatively charged molecules present in the environment, such as proteins, nucleic acids, or polysaccharides [17,23]. The electrostatic interactions between the positively charged hydrogel and the negatively charged molecules can be utilized in various applications [11,17]. For example, cationic polymer-based hydrogels have been explored for drug delivery systems, where the charged hydrogel can bind and deliver negatively charged drug molecules or interact with the negatively charged components of the biological environment. They have also found applications in wound healing and tissue engineering, where the interactions with negatively charged biomolecules play a role in promoting cellular adhesion and tissue engineering. On the other hand, anionic polymer-based hydrogels contain negatively charged functional groups such as carboxyl or sulfonate groups [206,216,217]. These hydrogels can interact with positively charged molecules, such as metal ions or proteins containing basic amino acid residues. The electrostatic interactions between the anionic hydrogel and the positively charged species can be harnessed for various applications [23,124,206]. The anionic polymer-based hydrogels have been investigated for drug delivery systems, where they can bind and release positively charged drugs or interact with positively charged proteins to modulate drug release [11,23]. They have also been explored in tissue engineering applications, where the interactions with positively charged molecules can influence cell behavior and tissue regeneration processes [174,217]. The presence of ionic charges in these hydrogels introduces unique properties and functionalities. The electrostatic interactions between the charged hydrogel and the surrounding environment can affect the swelling behavior, mechanical properties, and the release kinetics of encapsulated drugs or bioactive molecules. The ability to selectively interact with oppositely charged species provides opportunities for precise control over drug delivery, molecular recognition, and cellular interactions.

Ampholytic polymer-based hydrogels, also known as zwitterionic hydrogels, are a type of polymer-based hydrogel that contains both positively and negatively charged groups within the polymer chains [11,17,23,79]. These charged groups can be in the form of acidic and basic functional groups, such as carboxyl and amino groups. The presence of both positive and negative charges imparts amphoteric properties to the hydrogel, meaning it can exhibit acidic or basic behavior depending on the pH of the surrounding environment. The pH responsiveness of ampholytic polymer-based hydrogels arises from the ionization of the charged groups in response to changes in pH [218,219]. When the pH of the surrounding medium changes, the charged groups within the hydrogel can either gain or lose protons, leading to alterations in the overall charged density of the polymer-based hydrogel network [191,193,194]. This change in charged density affects the electrostatic interactions within the hydrogel and influences its swelling behavior and mechanical properties. As a result, ampholytic polymer-based hydrogels can undergo significant volume changes or alterations in their crosslinking density in response to pH variations [77,119,129]. The pH responsiveness of ampholytic polymer-based hydrogels makes them attractive for various applications. In drug delivery, these hydrogels can be designed to respond to specific pH conditions, such as those found in different regions of the body, to trigger drug release. For example, an ampholytic hydrogel with a lower critical solution temperature (LCST) above physiological pH can undergo a volume phase transition and release a loaded drug when exposed to acidic conditions, such as those in tumor microenvironments [76,77,218,219]. In tissue engineering, the pH responsiveness of ampholytic hydrogels can be exploited to modulate cell adhesion, proliferation, and differentiation by creating pH-tunable microenvironments that mimic native tissue conditions. Additionally, the ability of ampholytic hydrogels to interact with biomolecules due to their charged nature makes them suitable for biosensor applications, where they can selectively bind or detect specific analytes based on pH changes [78,119,130,131]. Ampholytic polymer-based hydrogels offer advantages such as enhanced stability, improved biocompatibility, and reduced nonspecific interactions compared to ionic hydrogels. The presence of both positive and negative charges in the hydrogel network can provide a balanced response to pH variations, allowing for precise control over their properties and behavior.

#### 3.1.4. Classification of Polymer-Based Hydrogels Based on Pore Size

Polymer-based hydrogels can exhibit different structural features, including pore size and interstitial domains. Pore size refers to the void spaces or cavities within the hydrogel structure that are interconnected and allow for the diffusion of solvents, gases, or other molecules [11,23]. Pores are typically defined as openings or channels that can be accessed by molecules, and their size is often characterized by the diameter of the opening. On the other hand, interstitial domains refer to the spaces between the polymer chains or networks within the hydrogel structure. These domains are not necessarily interconnected and may not provide direct pathways for molecule diffusion [17,220]. Instead, they contribute to the overall structure and integrity of the hydrogel. It is important to note that not all hydrogels exhibit well-defined or interconnected pores. Some hydrogels may have a more homogeneous structure with minimal or no discernible pores. In such cases, the transport of molecules occurs primarily through the interstitial domains, where the polymer chains create a network with sufficient space for solvent or molecule diffusion. The interstitial domains provide a pathway for molecular transport within the hydrogel, although they may not be explicitly characterized as “pores” due to their lack of well-defined openings or channels [11,17,23].

Polymer-based hydrogels can be engineered to have a specific pore size, which can influence their properties and applications. The presence of pores in polymer-based hydrogels is not a universal feature and depends on the specific manufacturing process used to produce the hydrogel. This means not all polymer-based hydrogels are necessarily porous, although all hydrogels are swellable [220,221]. Pores can be intentionally introduced during the hydrogel fabrication process through various methods such as solvent evaporation, freeze-drying, or templating techniques. These methods create void spaces or cavities within the hydrogel structure, resulting in a porous matrix [28,29,30,31,90,189]. Porous hydrogels have distinct advantages compared to non-porous hydrogels. The presence of pores provides increased surface area, which can enhance the diffusion of molecules, promote cell infiltration, and support tissue integration in biomedical applications [220,221]. Porous hydrogels can also facilitate the loading and release of drugs or bioactive agents, making them useful for drug delivery systems [89,202]. However, it is important to note that not all hydrogels require porosity to fulfill their intended functions. Many hydrogels are designed for applications where swelling and gel formation are the primary requirements, such as in contact lenses, wound dressings, or as matrices for tissue engineering scaffolds [28,29,30,31,89,90]. In these cases, the absence of pores does not hinder the performance of polymer-based hydrogels. The control of pore size in hydrogels is crucial for regulating the transport of molecules, cells, and fluids within the gel matrix. Here are three types of polymer-based hydrogels based on their pore size:

Macroporous polymer-based hydrogels are hydrogels that possess a three-dimensional network with relatively large, interconnected pores [90]. The macroporous structure allows for the easy flow of fluids, transport of cells, and tissue ingrowth. These hydrogels have pore sizes ranging from tens to hundreds of micrometers, providing ample space for the infiltration of cells, nutrients, and oxygen [11,23]. There are several methods to create macroporous structures in polymer-based hydrogels such as salt leaching, gas foaming, or solvent casting/particulate leaching. For the salt leaching technique, a water-soluble salt (e.g., sodium chloride) is mixed with the hydrogel precursor solution. After gelation, the salt particles create void spaces within the hydrogel matrix [11,17,90]. Subsequent leaching with water or a suitable solvent dissolves the salt particles, leaving behind a macroporous structure. Gas foaming involves the incorporation of a gas-generating agent (e.g., sodium bicarbonate) into the hydrogel precursor solution. When the hydrogel is crosslinked, the gas-generating agent releases gas bubbles, leading to the formation of macropores [11,23,90]. The bubbles can be stabilized by controlling the crosslinking process, resulting in a stable macroporous structure. For the solvent casting/particulate leaching method, a mixture of the hydrogel precursor and porogen particles (e.g., sugar particles or gelatin microspheres) is cast into a mold. After gelation, the porogen particles are selectively removed by leaching with a suitable solvent, leaving behind interconnected macropores. Macroporous polymer-based hydrogels have a wide range of applications, particularly in tissue engineering [11,17,90]. The large pore size facilitates cell infiltration, nutrient and oxygen transport, and the removal of waste products. The macroporous structure mimics the extracellular matrix, providing a favorable environment for cell adhesion, proliferation, and tissue regeneration [23,90]. These hydrogels can serve as scaffolds for various tissues, including bone, cartilage, skin, and vascular tissues. Additionally, macroporous hydrogels can be utilized in drug delivery systems, where the large pores enable the encapsulation and sustained release of therapeutic agents.

Mesoporous polymer-based hydrogels are a type of hydrogel that incorporate mesopores within their structure. Mesopores are defined as pores with diameters in the range of 2 to 50 nm [89,189]. These polymer-based hydrogels exhibit a high surface area and pore volume, providing unique properties and applications [17,23,89]. The synthesis of mesoporous polymer-based hydrogels typically involves the use of templating agents or self-assembly techniques with two commonly employed methods of template-assisted synthesis and self-assembly [11,189]. In templating agents or the self-assembly method, a sacrificial template, such as micelles or colloidal particles, is dispersed within the hydrogel precursor solution. After gelation, the template is selectively removed, leaving behind a network of interconnected mesopores [11,17,89]. The size and structure of the mesopores can be controlled by adjusting the size and characteristics of the template. On the other hand, self-assembly methods utilize the spontaneous organization of amphiphilic molecules or block copolymers to form mesostructures within the hydrogel matrix [23,189]. By controlling the self-assembly process, the formation of mesopores can be achieved. Hydrophilic–hydrophobic interactions and the arrangement of polymer chains dictate the mesopore structure [89,189]. The presence of mesopores in polymer-based hydrogels offers several advantages. The high surface area and pore volume allow for the efficient loading and release of molecules, making them promising for drug delivery systems. The mesoporous structure enables the controlled and sustained release of therapeutic agents [11,23]. Additionally, the mesopores can serve as reservoirs for bioactive molecules, growth factors, or cells, promoting tissue regeneration in tissue engineering applications. The properties and applications of mesoporous polymer-based hydrogels are diverse. They can be utilized for controlled drug release, biosensing, catalysis, and environmental remediation. The tailored mesoporous structure provides a platform for precise control over molecular diffusion, interactions, and reactions within the hydrogel matrix [11,17,23,189].

Microporous polymer-based hydrogels are a type of hydrogel that possess a network of interconnected micropores within their structure. Micropores are defined as pores with diameters typically less than 2 nm [23,202,221]. These hydrogels exhibit a high surface area and can absorb and retain a significant amount of fluid. The synthesis of microporous polymer-based hydrogels involves the introduction of porogens or porogenic agents during the polymerization process. These porogens are later removed, leaving behind a porous structure [11,17,23,221]. Various techniques can be employed to generate micropores, including solvent casting and particulate leaching and gas foaming. The solvent casting and particulate leaching method involves casting a mixture of polymer and porogenic particles, such as salt crystals or sugar particles, into a mold. After solidification, the porogens are dissolved or leached out, creating micropores in the hydrogel [11,23,202]. In the gas-foaming approach, a gas, such as nitrogen or carbon dioxide, is dissolved in the hydrogel precursor solution under high pressure. Upon rapid depressurization, the gas forms bubbles within the hydrogel, resulting in a microporous structure upon gelation [202,221]. Microporous polymer-based hydrogels offer several advantages due to their unique pore characteristics. The presence of micropores enhances the mechanical properties of the hydrogel, including its compressibility and flexibility [11,17,23]. The high surface area and porosity enable the efficient absorption and release of fluids, making them suitable for applications such as wound dressings, tissue engineering scaffolds, and biosensors [23,221]. These hydrogels can also be functionalized with specific chemical groups or bioactive molecules within the micropores, allowing for targeted drug delivery or the controlled release of therapeutic agents. The small pore size of microporous hydrogels can regulate the diffusion of molecules, providing tunable release profiles and prolonged drug delivery [11,202]. Furthermore, microporous polymer-based hydrogels can be designed to mimic the extracellular matrix (ECM) of biological tissues, facilitating cell adhesion, proliferation, and tissue regeneration. The micropores serve as pathways for cell migration, nutrient transport, and waste removal, promoting cell growth and tissue integration [11,17,23,202,221].

#### 3.1.5. Classification of Polymer-Based Hydrogels Based on Physical Appearance

Actually, the classification of polymer-based hydrogels based on physical appearance is unrelated to the polymerization technique. It refers to the different forms that the polymer-based hydrogels can take depending on their shape and structure. Here are the common classifications of polymer-based hydrogels based on their physical appearance:

Matrix polymer-based hydrogels, also known as bulk hydrogels, are three-dimensional networks composed of a polymer matrix that swells in the presence of water or biological fluids. These hydrogels are formed through the polymerization of monomers or the crosslinking of pre-polymers to create a continuous polymeric structure [40,51,198]. The polymer matrix provides structural integrity to the hydrogel while allowing for the absorption and retention of water within its network [73,73]. Matrix polymer-based hydrogels have a homogeneous structure throughout and occupy a relatively large volume. They can be soft and gel-like or have a more solid-like consistency, depending on the crosslinking density and composition of the polymer network [54,95,100]. The polymer chains within the hydrogel matrix are typically hydrophilic and contain hydrophilic functional groups, such as hydroxyl or amide groups, that facilitate water absorption. The properties of matrix polymer-based hydrogels can be tailored by selecting specific polymers, crosslinking methods, and polymerization conditions [185,189,198]. These hydrogels can exhibit various characteristics, including a high water content, biocompatibility, biodegradability, and mechanical properties that can range from soft and elastic to stiff and rigid [40,185]. The swelling behavior of matrix polymer-based hydrogels can be controlled by adjusting the crosslinking density and polymer composition. Matrix polymer-based hydrogels find a wide range of applications in areas such as tissue engineering, drug delivery, wound healing, and biosensing [40,51,73]. They can be used as scaffolds to support cell growth and tissue regeneration, as matrices for the controlled release of drugs or bioactive molecules, and as biomimetic materials to mimic the extracellular matrix. The versatility of matrix polymer-based hydrogels makes them valuable for developing advanced biomaterials for biomedical applications.

Film polymer-based hydrogels, also known as thin film, are hydrogel materials that are fabricated in form of thin films or coatings [64,90,188]. Unlike bulk hydrogels, which have a three-dimensional network structure, film hydrogels are typically two-dimensional and have a flat, sheet-like morphology. These hydrogels are composed of polymers that can absorb and retain water, similar to other hydrogel types [90,163]. Film polymer-based hydrogels are often prepared by casting, deposition techniques, or by using electrospinning techniques, where a solution or dispersion of hydrogel precursors is spread onto a substrate and then subjected to a crosslinking process to form a solid film [88,89]. The crosslinking can be achieved through various methods, such as chemical crosslinking, physical crosslinking, or photo-polymerization, depending on the specific polymer system used [64,163,188]. The thickness of film hydrogels can vary depending on the application requirements and the desired properties. They can range from a few micrometers to several millimeters in thickness. The composition of the polymer matrix can also be tailored to achieve specific properties such as mechanical strength, swelling behavior, and biocompatibility [23,64,90]. Film polymer-based hydrogels offer several advantages in various applications. Their thin and flexible nature allows for easy handling and conformability to different surfaces, making them suitable for coating medical devices, implants, or wound dressings. They can provide a protective barrier, promote moisture retention, and deliver bioactive substances to the target site. Film hydrogels also find applications in biosensing, where they can be utilized as thin films on sensor surfaces to detect specific analytes or biomarkers [11,17,163]. The film polymer-based hydrogels offer versatility and customization possibilities, making them attractive for a wide range of applications that require thin, flexible, and water-absorbent materials.

Microsphere polymer-based hydrogels are a type of hydrogel material that combine the properties of both microspheres and hydrogels [161,222]. These hydrogels are composed of small, spherical particles called microspheres that are made of a crosslinked polymer network. The microspheres are typically in the micrometer size range, ranging from a few micrometers to hundreds of micrometers in diameter [96,148]. The microspheres in microsphere polymer-based hydrogels can be made from a variety of polymers, such as natural polymers (e.g., gelatin or alginate) or synthetic polymers (e.g., polyvinyl alcohol or poly(lactic-co-glycolic acid)) [11,23,160]. These polymers are crosslinked to form a three-dimensional network within each microsphere, creating a porous structure. The pores within the microspheres can absorb and retain water or other aqueous solutions, giving them hydrogel-like properties [96,159,222]. Microsphere polymer-based hydrogels offer several advantages in different applications. The microspheres provide a high surface-area-to-volume ratio, allowing for the efficient encapsulation and delivery of bioactive molecules, such as drugs or growth factors [23,161]. The porous structure of the microspheres allows for the controlled release of the encapsulated substances, providing sustained and localized delivery over time. These hydrogels are also useful in tissue engineering and regenerative medicine [11,96,160]. The microspheres can serve as scaffolds or carriers for cells, facilitating their proliferation and differentiation. The porous structure of the microspheres allows for cell infiltration and nutrient exchange, supporting tissue growth and regeneration [96,148,161]. Microsphere polymer-based hydrogels can be prepared using various techniques, including emulsion methods, solvent evaporation, or spray drying. The size, porosity, and mechanical properties of the microspheres can be tailored by adjusting the polymer composition, crosslinking method, and processing parameters [17,222]. Microsphere polymer-based hydrogels provide a versatile platform for drug delivery, tissue engineering, and other biomedical applications [11,23,161]. Their unique combination of microsphere and hydrogel properties allows for precise control over drug release kinetics and cellular behavior, making them valuable in the development of advanced therapeutic and regenerative strategies.

Nanoparticle polymer-based hydrogels are hydrogel materials that incorporate nanoparticles within their polymer network [166,167,168]. These hydrogels combine the properties of both hydrogels and nanoparticles, offering unique characteristics and functionalities. The nanoparticles in nanoparticle polymer-based hydrogels can be either organic or inorganic in nature [77,78,82,150]. Examples of organic nanoparticles include polymeric nanoparticles, liposomes, micelles, or dendrimers, while inorganic nanoparticles may include metallic, magnetic, or quantum dot nanoparticles [14,38]. These nanoparticles are typically dispersed or embedded within the hydrogel matrix during the fabrication process. The presence of nanoparticles in the hydrogel network can impart several advantages to the material [20,42,64]. First, nanoparticles can enhance the mechanical properties of hydrogels, improving their strength, elasticity, and stability [62,124,126]. This is particularly important in applications where robustness is required, such as tissue engineering or drug delivery [153,163,166,167,168]. Second, the incorporation of nanoparticles can provide additional functionalities to the hydrogel [11,23]. For instance, magnetic nanoparticles can enable magnetic targeting or imaging capabilities, while fluorescent nanoparticles can facilitate the imaging and tracking of the hydrogel in biological systems [150,162]. Similarly, nanoparticles with specific surface chemistry can enable controlled drug release, enhance biocompatibility, or promote cellular interactions. Furthermore, nanoparticles within the hydrogel network can influence the release kinetics of encapsulated drugs or therapeutic agents [14,77,82]. The size, shape, and surface properties of the nanoparticles can affect the diffusion and release behavior, allowing for controlled and sustained drug delivery. Nanoparticle polymer-based hydrogels can be fabricated using various techniques, such as in situ polymerization, co-precipitation, self-assembly, or the surface modification of pre-synthesized nanoparticles followed by their incorporation into the hydrogel matrix [23,78,126]. The choice of fabrication method depends on the desired nanoparticle properties, compatibility with the hydrogel matrix, and the targeted application. Applications of nanoparticle polymer-based hydrogels span various fields. They have been used in drug delivery systems for the targeted and controlled release of therapeutics, as scaffolds for tissue engineering and regenerative medicine, as biosensors for the detection and monitoring of biomarkers, and in imaging and diagnostic technologies [23,150]. Nanoparticle polymer-based hydrogels offer a versatile platform that combines the unique properties of both nanoparticles and hydrogels. Their tunable properties, enhanced functionalities, and diverse applications make them attractive for a wide range of biomedical and technological advancements.

Polymer-based hydrogel beads, also known as gel beads, are small spherical particles made of hydrogel materials [180,223]. These beads are typically composed of crosslinked polymer networks that can absorb and retain large amounts of water or biological fluids. These hydrogel beads are often prepared through a process called suspension polymerization or emulsion polymerization. In this method, the monomers and crosslinking agents are dispersed in a continuous phase, such as water or an organic solvent, along with a surfactant or stabilizer to prevent aggregation. Polymerization is then initiated to form a three-dimensional network of interconnected polymer chains, resulting in the hydrogel structure of the beads [11,23,119,180]. The size of the beads can be controlled by adjusting the polymerization conditions, such as the concentration of monomers, crosslinking density, or stirring speed. Polymer-based hydrogel beads have various applications across different fields. In drug delivery, they can be used as carriers for the controlled and targeted release of drugs or therapeutic agents [17,223]. The porous structure of the beads allows for the encapsulation of drugs within the hydrogel matrix, and the release kinetics can be modulated by adjusting the polymer composition and crosslinking density. Additionally, the beads can be functionalized with specific ligands or targeting moieties to enhance their binding affinity to specific cells or tissues [11,180]. In biotechnology and diagnostics, polymer-based hydrogel beads are used for applications such as enzyme immobilization, protein separation, cell encapsulation, and biosensing [11,17,119]. The large surface area and high porosity of the beads facilitate efficient enzyme loading or protein binding, enabling enhanced catalytic activity or the effective separation of biomolecules. They can also serve as a matrix for encapsulating cells in tissue engineering or regenerative medicine applications [23,180]. Polymer-based hydrogel beads can also find use in cosmetics, agriculture, and environmental engineering. For example, they can be employed in personal care products for the controlled release of active ingredients or as absorbent materials for moisture management [23]. In agriculture, hydrogel beads can improve water retention in soil and facilitate the controlled release of fertilizers [11]. In environmental applications, they can be utilized for water treatment, the adsorption of pollutants, or controlled release of chemicals for environmental remediation [11,23].

There are other physical appearances that hydrogels can take, depending on the manufacturing technique and intended application.

#### 3.1.6. Classification of Polymer-Based Hydrogels Based on Crystallinity

Polymer-based hydrogels can be classified based on their crystallinity, which relates to the degree of structural order in the polymer network. Crystallinity influences the physical and mechanical properties of hydrogels, including their strength, stiffness, and swelling behavior. In terms of crystallinity, hydrogels can be broadly categorized into two types, with amorphous polymer-based hydrogels [224,225] and semi-crystalline polymer-based hydrogels [17,23,226].

Amorphous polymer-based hydrogels are hydrogels that lack long-range structural order or crystallinity in their polymer network [23,224,225]. The polymer chains in these hydrogels are randomly distributed, resulting in a more disordered and non-crystalline structure [224]. Amorphous polymer-based hydrogels are characterized by the absence of well-defined crystalline domains, which distinguishes them from crystalline hydrogels [225]. Without the presence of ordered polymer segments, amorphous hydrogels exhibit different physical and mechanical properties [23,224]. Amorphous polymer-based hydrogels find applications in various fields, including drug delivery systems, wound dressings, tissue engineering scaffolds, and biosensors [11,23]. Their flexibility and high water absorption capacity make them suitable for conformal contact with biological tissues, while their transparency and biocompatibility are advantageous for biomedical applications [11,23,225]. It is worth noting that the distinction between amorphous and crystalline hydrogels is not always absolute, as some hydrogels may exhibit a combination of amorphous and crystalline regions to varying degrees [224]. The degree of crystallinity in a hydrogel can be influenced by factors such as the choice of polymer, synthesis conditions, and post-processing treatments [23,225].

Semi-crystalline polymer-based hydrogels are hydrogels that exhibit a combination of amorphous and crystalline regions within their polymer network [11,23,226]. Unlike amorphous polymer-based hydrogels that lack long-range structural order, semi-crystalline polymer-based hydrogels contain regions where the polymer chains arrange themselves in an ordered, crystalline manner, interspersed with amorphous regions [11,17]. The presence of crystalline regions in semi-crystalline hydrogels introduces additional structural organization and can significantly influence the material properties [226]. The development of semi-crystalline polymer-based hydrogels involves careful control of the synthesis and processing conditions to promote the formation of crystalline regions [23]. Factors such as polymer selection, crystallization temperature, and annealing can influence the degree of crystallinity within the hydrogel [23,226]. The unique combination of crystalline and amorphous regions in semi-crystalline hydrogels offers advantages in various applications. For instance, their enhanced mechanical properties make them suitable for load-bearing tissue engineering scaffolds [11,17], while their thermal stability is advantageous for applications requiring exposure to higher temperatures [17,23]. The balance between crystallinity and amorphousness can be tailored to achieve the desired properties for specific applications [11,23].

The degree of crystallinity in a polymer-based hydrogel can be controlled by adjusting the polymerization conditions, such as temperature, pH, and the concentration of the polymer and crosslinking agent [23,226].

#### 3.1.7. Classification of Polymer-Based Hydrogels Based on Crosslinking

Polymer-based hydrogels can indeed be classified based on the type of crosslinking they employ. The crosslinking mechanism is crucial in determining the structure, properties, and performance of the hydrogel. The two main types of crosslinking in polymer-based hydrogels are physical crosslinking [58,227,228] and chemical crosslinking [23,58,173,228].

Chemical crosslinking involves the formation of covalent bonds between the polymer chains, resulting in a three-dimensional network structure [23,228]. Chemical crosslinking methods include radical polymerization, Michael addition, Schiff base reaction, and epoxy crosslinking, among others [58,173]. This process typically requires the use of crosslinking agents or the incorporation of functional groups in the polymer chains that can react and form covalent bonds [228]. Chemical crosslinking reactions can be initiated by various methods such as heat, light, or chemical initiators [11,23]. Once the crosslinks are formed, they are permanent and stable. Chemically crosslinked hydrogels often exhibit excellent mechanical strength, stability, and resistance to dissolution [58,173]. However, the crosslinking process may involve harsh conditions or toxic chemicals, which can limit their applications in certain sensitive environments or in biomedical applications [11,23,228].

Physical crosslinking relies on reversible, non-covalent interactions between polymer chains to form a network structure [227,228]. These interactions can include hydrogen bonding, hydrophobic interactions, electrostatic interactions, or the physical entanglement of polymer chains [11,23]. Physical crosslinking methods offer advantages such as mild processing conditions and the ability to tune the network structure and properties by altering the environmental conditions [17,227]. Common physical crosslinking methods include temperature-induced gelation, pH-induced gelation, ion-induced gelation, and self-assembly, among others [23,228]. Physically crosslinked hydrogels can exhibit stimuli-responsive behavior, such as swelling/deswelling in response to environmental changes, due to the reversible nature of the interactions [228]. However, physically crosslinked hydrogels generally have lower mechanical strength compared to chemically crosslinked hydrogels and can be sensitive to changes in environmental conditions [227,228].

The choice between chemical and physical crosslinking depends on the desired properties and applications of the hydrogel. Chemically crosslinked hydrogels are often preferred when high mechanical strength, stability, and resistance to degradation are required [11,23]. They are commonly used in load-bearing applications, tissue engineering scaffolds, and long-term drug delivery systems. On the other hand, physically crosslinked hydrogels are advantageous when stimuli-responsive behavior, injectability, or biodegradability are desired [23,58,227,228]. They find applications in drug delivery, tissue engineering, and regenerative medicine, where the ability to respond to specific triggers or undergo controlled degradation is beneficial.

#### 3.1.8. Classification of Polymer-Based Hydrogels Based on External Stimuli Response

Polymer-based hydrogels can be classified based on their responsiveness to external stimuli, which is an important characteristic that enables their use in various applications. These stimuli-responsive polymer-based hydrogels, also known as smart hydrogels or intelligent hydrogels, exhibit changes in their structure, properties, or behavior in response to specific external stimuli [11,23]. This responsiveness is a result of the specific design and incorporation of responsive components or functional groups within the hydrogel network.

Temperature-responsive polymer-based hydrogels are a type of hydrogel that exhibit changes in their swelling behavior or sol–gel transition in response to changes in temperature [74,75,131,192,193]. These hydrogels are designed to undergo a volume phase transition at a specific temperature, known as the lower critical solution temperature (LCST) or an upper critical solution temperature (UCST) [23,192,193]. The transition can be reversible or irreversible, depending on the specific polymer and formulation used. Temperature-responsive polymer-based hydrogels typically consist of a polymer network that incorporates thermo-responsive components, such as N-isopropylacrylamide (NIPAAm), poly(N-isopropylacrylamide) (PNIPAAm), or polyethylene glycol (PEG). These polymers have LCSTs close to physiological temperatures, making them suitable for biomedical applications [23,74,75]. Below the LCST, temperature-responsive polymer-based hydrogels are in a swollen state, allowing for the absorption and retention of water. As the temperature increases beyond the LCST, the hydrogel undergoes a phase transition and collapses, resulting in a decrease in swelling and an increase in mechanical strength [17,131]. The LCST of temperature-responsive hydrogels can be adjusted by varying the polymer composition, molecular weight, and crosslinking density. This allows for the fine-tuning of the hydrogel properties to match specific application requirements. Temperature-responsive polymer-based hydrogels have a wide range of applications, particularly in drug delivery systems and tissue engineering [11,23,192,193]. They can be used as carriers for controlled drug release, where the drug is released at a higher rate above the LCST due to the collapse of the hydrogel. In tissue engineering, these hydrogels can serve as scaffolds for cell culture, where the ability to switch between a swollen and collapsed state can mimic dynamic cellular environments and facilitate cell attachment, proliferation, and differentiation [17,23,74,75].

pH-responsive polymer-based hydrogels are a type of hydrogel that exhibit changes in their swelling behavior or properties in response to variations in pH [76,77,78,129,130,131]. These hydrogels can contain pH-sensitive functional groups within their polymer networks, allowing them to respond to acidic or basic conditions. The pH-responsive behavior of these hydrogels arises from the ionization or deionization of the functional groups within the polymer network as the pH of the surrounding environment changes [119,120]. Common pH-sensitive functional groups used in the design of pH-responsive hydrogels include carboxylic acid (COOH), amino groups (NH_2_), and imidazole groups (imidazole). At certain pH values, these functional groups can either be ionized or deionized, leading to changes in the charge density and hydrophilicity of the hydrogel. This, in turn, affects the swelling or collapse of the hydrogel network [119,120]. For example, in an acidic environment (low pH), hydrogels containing ionizable carboxylic acid groups will be in a collapsed or shrunken state due to increased protonation of the acidic groups. As the pH increases towards neutrality or becomes basic, the carboxylic acid groups deprotonate, leading to an increase in hydrogel swelling [191,193,194]. The pH-responsive behavior of these hydrogels can be fine-tuned by modifying the type and concentration of the pH-sensitive functional groups, as well as the crosslinking density of the polymer network [218,219]. This allows for precise control over the pH range at which the hydrogel exhibits responsive behavior. pH-responsive polymer-based hydrogels have numerous applications, particularly in drug delivery systems and biosensors. In drug delivery, these hydrogels can be used to release drugs in a pH-dependent manner, where the hydrogel swells or collapses in response to the pH of the target site, leading to controlled release kinetics [129,130,131]. In biosensors, pH-responsive polymer-based hydrogels can serve as sensing elements that undergo changes in their optical or electrical properties in response to pH variations, enabling the detection of pH-related analytes or environmental changes [76,77,78]. The pH-responsive nature of these hydrogels makes them highly versatile and valuable in biomedical and biotechnological applications, providing a platform for responsive materials that can be tailored to the specific pH conditions encountered in biological systems.

Light-responsive polymer-based hydrogels, also known as photoresponsive or photosensitive hydrogels, are a type of hydrogel that can undergo changes in their properties or behavior upon exposure to light [121,122,123]. These hydrogels contain light-sensitive components or photoactive molecules that can be activated or deactivated by specific wavelengths of light [121]. Light-responsive polymer-based hydrogels can be classified into two main categories of photothermal-responsive polymer-based hydrogels and photoresponsive polymer-based hydrogels [122]. The first one exhibits a response to light in the form of heat generation. They are typically composed of polymers or nanoparticles that can convert light energy into heat through processes such as plasmonic absorption or photothermal conversion [123]. The heat generated can trigger changes in the swelling, sol–gel transition, or mechanical properties of the hydrogel. The second one undergoes changes in their structure, shape, or properties upon exposure to light, without involving heat generation [23]. This response is achieved through the incorporation of photoresponsive molecules, such as photochromic compounds or light-sensitive crosslinkers, into the polymer network [11]. These molecules undergo reversible photoisomerization or photodimerization reactions when illuminated with specific wavelengths of light, leading to conformational changes or crosslinking/de-crosslinking of the polymer network [23,122]. Light-responsive polymer-based hydrogels offer precise spatiotemporal control over their properties and functionalities, making them valuable in various applications such as drug delivery systems, tissue engineering, actuators and soft robotics, and optical sensors and imaging [121,122,123]. The choice of photoactive molecules and the design of the hydrogel network can be tailored to achieve the desired light responsiveness, such as specific light wavelengths, intensity, and response kinetics. This versatility allows for the development of smart materials with controlled and tunable photoresponsive properties for a wide range of applications [11,23,121,122,123].

Electric-field-responsive polymer-based hydrogels, also known as electroactive hydrogels, are a type of hydrogel that can undergo changes in their properties or behavior in the presence of an electric field [229,230]. These hydrogels contain electrically conductive components or responsive polymers that can be influenced by the application of an electric field [30,216]. The response of electric-field-responsive hydrogels can be attributed to several underlying mechanisms of electromechanical effect, electroosmosis, and electrically triggered release [70,229]. The response of electric-field-responsive hydrogels can be controlled by adjusting parameters such as the electric field strength, frequency, or waveform [216,230]. This allows for the precise modulation of the hydrogel’s behavior and functionality. Electric-field-responsive hydrogels find applications in various fields such as tissue engineering, sensors and actuators, and drug delivery [229,230]. The specific design and composition of electric-field-responsive hydrogels can vary depending on the desired application and the targeted response [30,70]. By integrating electrically responsive components into the hydrogel matrix, these materials offer unique opportunities for the development of advanced functional systems with electrically tunable properties [23,216,229,230].

Magnetic-field-responsive polymer-based hydrogels are a type of hydrogel that can exhibit changes in in their properties or behavior in the presence of a magnetic fields [126,127,128]. These hydrogels contain magnetic particles or responsive polymers that can be influenced by magnetic forces [222,223]. The response of magnetic-field-responsive hydrogels is attributed to the interaction between the embedded magnetic components and the applied magnetic field [126,222]. The magnetic particles or polymers within the hydrogel can respond to the magnetic field in several ways including magnetic actuation, thermal effects, and magnetic resonance imaging (MRI) contrast agents [127,223]. The response of magnetic-field-responsive hydrogels can be tuned by adjusting parameters such as the strength and orientation of the magnetic field, as well as the concentration and properties of the magnetic components within the hydrogel [126,127,128,223]. They have potential applications in drug delivery, tissue engineering, biomedical devices, and magnetic resonance imaging [222,223]. Magnetic-field-responsive polymer-based hydrogels offer unique advantages in terms of remote control, non-invasiveness, and tunability. Their ability to respond to magnetic fields opens up opportunities for various biomedical applications where external manipulation or control is desired [126,127,128,222,223].

Classifications of polymer-based hydrogels are listed in Table 3 as follows:

These classifications are not mutually exclusive, and a hydrogel can belong to more than one category depending on its properties and composition.

### 3.2. Main Engineering Properties of Polymer-Based Hydrogels’ Influence on DDSs

#### 3.2.1. Swelling Property with Most Important Role in DDSs

Swelling is one of the most important characteristics of polymer-based hydrogels. Swelling in polymer-based hydrogels occurs when a solvent permeates and diffuses through the interstitial space within the hydrogel network [5,24,25,26]. The interstitial space is defined by the arrangement and density of the polymer chains, which are determined by the crosslinking process kinetics and conditions [45,55]. Several metrics are commonly used to quantify the characteristics of the interstitial space in a polymer-based hydrogel network [120,130]. The first is the molecular weight between crosslinks, referring to the average distance between two adjacent crosslinks along a polymer chain. A higher molecular weight between crosslinks corresponds to a larger distance, resulting in a more extended polymer network and larger interstitial spaces [169,170,171]. The second is mesh size, which represents the average distance between adjacent polymer chains within the hydrogel network. It determines the size of the gaps or voids available for solvent penetration [5,23]. A larger mesh size indicates larger interstitial spaces and higher swelling capacity. The last one is crosslink density where the number of crosslinks per unit volume in the hydrogel network is quantified [11,55]. A lower crosslink density leads to a more loosely packed polymer network, creating larger interstitial spaces and promoting higher swelling capabilities [17,120]. These metrics play a crucial role in understanding and predicting the swelling behavior of hydrogels [23]. By manipulating the crosslinking conditions, such as polymer concentration, crosslinker concentration, and reaction time, it is possible to control the interstitial space and subsequently the swelling properties of the polymer-based hydrogel [11,23,169,170,171].

Polymer-based hydrogels have a unique ability to absorb and retain large amounts of water due to the presence of hydrophilic groups in their polymer chains [5,23]. The swelling behavior of hydrogels is influenced by several factors, such as the chemical composition of the hydrogel, crosslinking density, and the properties of the solvent. It can be described by parameters such as equilibrium swelling ratio (Q), swelling kinetics, and diffusion coefficients [11,120].

(1) $$Q=\frac{\mathrm{Ws}-W_{d}}{W_{d}}$$

W_s_ is the weight of the swollen hydrogel.

W_d_ is the hydrogel’s weight before swelling with water.

The equilibrium swelling ratio (Q) is a parameter that quantifies the extent of swelling exhibited by a polymer-based hydrogel. It is defined as the ratio of the weight or swollen volume of the hydrogel to its dry weight or volume in equilibrium with the surrounding solvent [23,24,25,26]. Q is a function of the crosslinking density, degree of ionization of the functional groups, temperature, and pH of the surrounding medium. Q provides information about the capacity of the hydrogel to absorb and retain water, and it is influenced by the polymer structure, crosslinking density, and interactions with the solvent [45,55].

Swelling kinetics refers to the time-dependent process of hydrogel swelling. It involves the absorption of solvent molecules into the hydrogel network and the subsequent equilibrium establishment [11,45]. The kinetics can be influenced by factors such as the diffusivity of the solvent, the presence of barriers or hindrances within the hydrogel structure, and the availability of swelling sites [23,130]. Understanding the swelling kinetics is important for optimizing hydrogel performance in applications where controlled swelling or the release of substances is desired [24,25,26,169,170,171].

Diffusion coefficients are parameters that describe the rate at which solutes can diffuse within the hydrogel network. They provide information about the mobility of solute molecules within the hydrogel and their ability to traverse the polymer chains or interstitial spaces [11,23,24,25,26]. Diffusion coefficients are affected by the porosity of the hydrogel, polymer chain density, and interactions between the solute and the hydrogel matrix [45,45]. Knowledge of diffusion coefficients is essential for designing the hydrogels used in drug delivery systems, as it influences the release kinetics of encapsulated substances.

The swelling properties of hydrogels make them attractive for various applications such as drug delivery, tissue engineering, and biosensors [11,23]. In drug delivery, the swelling properties of hydrogels can be utilized to control the release of drugs by adjusting the crosslinking density and the degree of ionization of the functional groups [23,24,25,26,120]. In tissue engineering, hydrogels can provide a three-dimensional matrix for cell growth and proliferation due to their ability to mimic the extracellular matrix of natural tissues [169,170,171]. In biosensors, hydrogels can be used as a transducer to convert biological signals into measurable signals by incorporating enzymes or antibodies into the hydrogel network [5,11,120,130]. There are several factors that can affect the swelling properties of hydrogels, including:

Chemical composition: The chemical composition of the hydrogel polymer can affect the swelling properties. Hydrogels with more hydrophilic groups (such as hydroxyl, amide, or carboxyl groups) tend to have higher swelling ratios than those with fewer hydrophilic groups [5,23].

Crosslinking density: The degree of crosslinking of the hydrogel network affects the swelling properties. Hydrogels with a higher crosslinking density tend to have a lower swelling ratio, as there are fewer available spaces for water molecules to enter and swell the hydrogels [11,23,24,25,26].

pH and ionic strength: Changes in the pH and ionic strength of the surrounding environment can affect the swelling properties of hydrogels. For example, acidic environments can protonate amine groups on the polymer chains, leading to a decrease in swelling. On the other hand, basic environments can deprotonate carboxylic acid groups on the polymer chains, leading to an increase in swelling [11,17,23,169,170,171].

Temperature: Temperature can affect the swelling properties of some hydrogels. For example, some thermo-responsive hydrogels can swell or shrink in response to changes in temperature [5,23,24,25,26].

Presence of other molecules: The presence of other molecules in the surrounding environment, such as salts or other solvents, can affect the swelling properties of hydrogels. These molecules can compete with water molecules for space within the hydrogel network, leading to changes in swelling [5,11,17].

#### 3.2.2. Permeation Property in Biomedical Applications

The permeation property of polymer-based hydrogels refers to their ability to allow the transport of molecules and ions through their three-dimensional network structure [23,166]. Polymer-based hydrogels can exhibit different permeation characteristics depending on their composition, crosslinking density, pore size, and other factors [11,48]. Understanding and controlling the permeation properties of hydrogels are crucial for their applications in fields such as drug delivery, tissue engineering, and biosensing [23,172]. The permeation behavior of polymer-based hydrogels is influenced by several factors, such as molecular size, pore size, crosslinking density, and chemical composition [11,17,23,172].

Molecular size: Hydrogels can selectively permeate molecules based on their size. Smaller molecules, such as gases, ions, and small hydrophilic compounds, can diffuse more easily through the hydrogel network [23]. In contrast, larger molecules, such as proteins or macromolecules, face greater resistance and may have limited permeability [11,23,48].

Pore size: The pores are characteristic of the porous polymer-based hydrogels. This is to make it clear that not all polymer-based hydrogels are porous. With the porous polymer-based hydrogels, it is the size of the pores which plays a significant role in determining the permeation properties [11,23]. Hydrogels with larger pore sizes generally exhibit higher permeability, allowing for the easier passage of molecules through the network [166,172]. The pore size can be controlled by adjusting the crosslinking density or by incorporating porogens during hydrogel synthesis [23,48].

Crosslinking density: The degree of crosslinking within the hydrogel network can affect permeation. A higher crosslinking density can lead to smaller pore sizes and reduced permeability, while lower crosslinking density can result in larger pores and increased permeability [11,23,166].

Chemical Composition: The chemical composition of the hydrogel, including the polymer type and functional groups, can influence permeation. Hydrogels made from polymers with charged functional groups may interact with charged molecules, affecting their permeation. Additionally, the presence of specific functional groups can be utilized to design hydrogels with selective permeation properties for targeted applications [11,23,172].

Controlling the permeation properties of polymer-based hydrogels allows for the design of materials with specific transport characteristics [23,48]. For example, hydrogels can be engineered to selectively allow the passage of certain molecules while restricting the permeation of others [48,172]. This control is essential in applications such as drug delivery, where controlled release kinetics and targeted delivery are desired. In tissue engineering, permeable hydrogels can facilitate nutrient and oxygen transport to support cell growth and viability [11,23,166]. The permeation properties of hydrogels can be tailored to meet specific needs for drug delivery, tissue engineering, and other applications.

#### 3.2.3. Mechanical Properties with a Crucial Role in Drug Delivery Systems

The mechanical properties of polymer-based hydrogels play a crucial role in drug delivery systems. These properties determine the stability, integrity, and functionality of the hydrogel as it interacts with biological tissues and releases drugs in a controlled manner [38,174,190,191,231]. Several key mechanical properties of hydrogels are relevant to drug delivery systems.

Elasticity and flexibility: The elasticity and flexibility of hydrogels influences their ability to conform to the surrounding tissue and withstand mechanical stresses [190,191,231]. Hydrogels with appropriate elasticity can adhere to irregular surfaces, such as wound sites, or be implanted in specific locations. This property ensures good contact with the target site and prevents the premature degradation or disintegration of the hydrogel [23,38,231].

Chemical composition: The chemical composition of the hydrogel can also affect its mechanical properties [11,190,191]. For example, hydrogels made from poly(acrylic acid) are generally brittle and have poor mechanical strength [232] while hydrogels made from poly(ethylene glycol) are more elastic and flexible [205]. Additionally, the presence of functional groups, such as double bonds, can also enhance the mechanical properties of hydrogels [23,231].

Swelling behavior: The swelling behavior of hydrogels is important in drug delivery systems. Swelling affects the release rate and diffusion of drugs from the hydrogel matrix. Controlled swelling can provide sustained release over an extended period [11,23]. Additionally, the extent of swelling can impact the mechanical stability of the hydrogel, affecting its ability to retain shape and integrity during drug release [17,231].

Viscoelasticity: The viscoelastic nature of hydrogels refers to their ability to exhibit both elastic and viscous behavior under mechanical stress [11]. Viscoelasticity is relevant in drug delivery systems as it affects the hydrogel’s ability to absorb and dissipate mechanical forces [23]. A suitable balance of elasticity and viscosity allows hydrogels to accommodate mechanical deformations, such as tissue movements or compression, without compromising their structural integrity [23,191].

Adhesion and cohesion: Adhesion and cohesion properties influence the interaction between the hydrogel and the surrounding tissue [11,17]. Adequate adhesion promotes close contact between the hydrogel and the target tissue, enhancing drug delivery efficiency. Cohesion is essential to maintain the integrity of the hydrogel and prevent drug leakage or migration [23,231].

The mechanical strength of hydrogels determines their ability to maintain structural integrity under mechanical forces. Hydrogels should possess sufficient strength to withstand handling during fabrication, implantation, and drug release [17,38]. Mechanical strength prevents structural failure and ensures the hydrogel remains intact throughout the drug delivery process [174]. The mechanical properties of polymer-based hydrogels can be tailored through various strategies, including adjusting the polymer composition, crosslinking density, and incorporating reinforcing materials or fillers [190,191]. By carefully controlling these mechanical properties, drug delivery systems can be optimized to achieve the desired drug release kinetics, improve tissue compatibility, and enhance therapeutic efficacy [23,191]. It is important to note that the mechanical properties of hydrogels used in drug delivery systems should be carefully optimized to ensure they meet the specific requirements of the application [231]. For instance, hydrogels for implantable devices or injectable systems may have different mechanical considerations than hydrogels used in topical or transdermal drug delivery [17]. Therefore, a thorough understanding of the mechanical behavior and requirements of the drug delivery system is crucial for a successful translation and implementation in biomedical applications.

#### 3.2.4. Biological Properties of Polymer-Based Hydrogels for DDSs

Polymer-based hydrogels used in drug delivery systems possess various biological properties that are important for their performance and effectiveness [3,8]. These properties enable hydrogels to interact with biological systems and facilitate controlled drug release [11,12]. Some key biological properties of polymer-based hydrogels for drug delivery systems include the following:

Biocompatibility refers to the ability of a material to coexist with living tissues without causing adverse reactions or toxicity [41,233]. Polymer-based hydrogels intended for drug delivery systems should exhibit high biocompatibility to minimize immune responses, inflammation, or tissue damage upon implantation or administration [101]. Biocompatible hydrogels ensure safe and well-tolerated drug delivery within the body [102].

Bioadhesion is the ability of hydrogels to adhere to biological tissues or surfaces. This property is particularly relevant for local drug delivery applications, where the hydrogel needs to adhere to the target site, such as wounds or mucosal membranes [23,233]. Bioadhesive hydrogels can prolong drug residence time, enhance drug penetration into tissues, and provide sustained drug release at the site of action [101,102].

Cell interaction and the tissue integration of polymer-based hydrogels meet in certain drug delivery applications, such as tissue engineering or regenerative medicine, where hydrogels are designed to interact with cells and support tissue growth and integration [234]. Hydrogels with appropriate biological properties can promote cell adhesion, proliferation, and differentiation, facilitating tissue regeneration and healing processes [235].

Controlled biodegradability is an important property for polymer-based hydrogels in drug delivery systems, as it allows for the gradual degradation and clearance of the hydrogel after drug release [2,11]. Controlled biodegradability ensures that the hydrogel is metabolized or eliminated from the body without causing harm or leaving residues. The degradation rate can be tailored to match the desired drug release kinetics and the tissue healing timeline [23,66].

The immunomodulation of polymer-based hydrogels influences the immune response at the site of drug delivery [11]. Depending on the application, hydrogels can be designed to stimulate or suppress immune reactions to optimize therapeutic outcomes. Immunomodulatory hydrogels can help promote tissue healing, prevent immune rejection, or enhance the effectiveness of immunotherapeutic drugs [17,23].

Drug stability and protection: Polymer-based hydrogels can provide protection to encapsulated drugs from degradation or inactivation [12,125]. They can shield drugs from enzymatic degradation, pH variations, or other harsh conditions in the body, preserving their stability and therapeutic activity until release [80,129]. This property is particularly important for sensitive drugs that require protection during transit to the target site [11,23].

By considering these biological properties, polymer-based hydrogels can be tailored to achieve an optimal drug delivery performance, ensuring compatibility with biological systems, the controlled release of therapeutic agents, and minimization of adverse effects [41]. The selection of appropriate polymer materials, fabrication techniques, and formulation strategies plays a crucial role in achieving the desired biological properties for drug delivery systems [23,101,102].

### 3.3. Applications of Polymer-Based Hydrogels in Drug Delivery Systems

Polymer-based hydrogels have been widely investigated for drug delivery applications due to their unique properties such as a high water content, biocompatibility, and the ability to respond to various stimuli [11,23]. Some of the applications of hydrogels in drug delivery systems include the following:

Controlled drug release: Polymer-based hydrogels can be used to control the release of drugs by varying the swelling behavior of the hydrogel. By altering the chemical composition or crosslinking density of the hydrogel, the release rate of the drug can be controlled [29,134].

Targeted drug delivery: Polymer-based hydrogels can be designed to specifically target certain tissues or cells. By incorporating targeting moieties such as antibodies or peptides into the hydrogel, the drug can be delivered to a specific site within the body [16,105].

Oral drug delivery: Polymer-based hydrogels can be used to improve the oral bioavailability of drugs. By encapsulating the drug within a hydrogel matrix, the drug can be protected from degradation in the gastrointestinal tract and released in a controlled manner [8,9,16].

Transdermal drug delivery: Polymer-based hydrogels can be used for transdermal drug delivery by incorporating the drug into the hydrogel matrix. The hydrogel can then be applied topically to the skin, and the drug will be released in a controlled manner over time [136,236].

Implantable drug delivery systems: Polymer-based hydrogels can be used as implantable drug delivery systems, where the hydrogel is placed within the body and releases the drug over an extended period of time. These systems can be used for the treatment of chronic diseases or for the delivery of long-acting drugs [69,108].

Gene delivery: Polymer-based hydrogels can also be used for the delivery of genetic material such as DNA or RNA. By incorporating the genetic material into the hydrogel matrix, the material can be protected from degradation and delivered to specific cells or tissues [115,237].

The unique properties of polymer-based hydrogels make them a promising candidate for drug delivery applications, and ongoing research in this area is expected to lead to the development of new and innovative drug delivery systems. There are common hydrogel-based products known as biotene, Simpurity™ Hydrogel (SupremeMed, Van Nuys City, City of Los Angeles, CA, USA), Soflens daily disposable, Nicorette^®^( Johnson & Johnson, Stockholm, Sweden), Neutrogena^®^ Hydro Boost^®^ (Johnson & Johnson, City of Los Angeles, CA, USA), Sericin (Huzhou Shengtao Biotech, Zhejiang, China), Suprasorb^®^ G (Lohmann & Rauscher, Rengsdorf, Germany) and others.

#### 3.3.1. Biotene Products

Biotene is an oral moisturizing agent that is commonly used for treating dry mouth. The product is manufactured by GlaxoSmithKline (London, United Kingdom) and contains a mixture of ingredients, including carbomer and hydroxyethyl cellulose, which are hydrogels that have the ability to absorb water and retain it within their three-dimensional structures. These hydrogels provide a sustained release of moisture in the oral cavity, relieving the symptoms of dry mouth and promoting oral health. Biotene is also available in other formulations, including mouthwash, toothpaste, and oral gel, and is recommended for individuals with dry mouth caused by medication, radiation therapy, or Sjogren’s syndrome [57].

#### 3.3.2. Simpurity^TM^ Hydrogel

Simpurity™ (from SupremeMed, Van Nuys City, City of Los Angeles, United States) Hydrogel from Safe n’ Simple is a hydrogel product that is used to treat skin burns, dry wounds, and dry scabs. The product is made from a combination of polyethylene oxide (PEO), polyvinyl alcohol (PVA), acrylate, polyurethane, and pure water to create absorbent sheets. The hydrogel is designed to provide a moist environment for wound healing and can also help to reduce pain and inflammation. It is a widely used product for wound care and is known for its effectiveness in promoting wound healing [232].

#### 3.3.3. Soflens Daily Disposable

Soflens daily disposable from Bausch & Lomb (New York, NY, USA) is a contact lens product used to correct short- and long-sightedness. The hydrogel material used in this product allows for a high water content and oxygen permeability, making the lenses comfortable for extended wear. The hydrogel also helps to maintain moisture around the lens, preventing dryness and discomfort for the wearer [194].

#### 3.3.4. Nicorette^®^ Hydrogel

Nicorette^®^ hydrogel from GlaxoSmithKline (London, UK) is a chewing gum used as a smoking cessation aid. It contains hydroxypropyl methylcellulose as one of its components. The hydrogel in the chewing gum acts as a carrier for the nicotine and helps to release it slowly over time, providing a controlled release of nicotine to help with cravings and withdrawal symptoms during the quitting process [57].

#### 3.3.5. Neutrogena^®^ Hydro Boost^®^ Hydrogel

Neutrogena^®^ Hydro Boost^®^ is a hydrogel-based face mask from Johnson and Johnson (City of Los Angeles, CA, USA)) that is used to provide the skin with immediate and long-lasting moisture. The key ingredient in the mask is hyaluronic acid, which is a naturally occurring polysaccharide that can hold up to 1000 times its weight in water. The hydrogel mask is designed to adhere to the skin and slowly release the hydrating ingredients over time, leaving the skin feeling soft, supple, and moisturized [85].

#### 3.3.6. Sericin Hydrogel

Sericin (Huzhou Shengtao Biotech, Zhejiang, China) has also been investigated for its potential use in drug delivery systems. In one study, sericin was used as a carrier for an optically trackable drug delivery system for malignant melanoma. The sericin nanoparticles loaded with the drug were shown to effectively deliver the drug to cancer cells and inhibit their growth, indicating its potential as a promising drug delivery system for cancer therapy [238].

#### 3.3.7. Suprasorb^®^ G Hydrogel

Suprasorb G (Lohmann & Rauscher, Rengsdorf, Germany) hydrogel is a wound dressing that is used for the management of various types of wounds such as lower leg ulcers, pressure ulcers, first- and second-degree burns, and scalds. The hydrogel is composed of a blend of acrylic polymers, polyethylene (PE), and phenoxyethanol, and contains 70% water. The hydrogel provides a moist environment for the wound, which promotes wound healing by facilitating the migration of cells and promoting the formation of granulation tissue. The water content of the hydrogel also helps to cool the wound, reducing pain and inflammation. Suprasorb G hydrogel is easy to apply and remove and can be used on both dry and exuding wounds [46].

### 3.4. Current and Future Trends of Research on Polymer-Based Hydrogels Applied in Drug Delivery Systems

**Figure 2:**
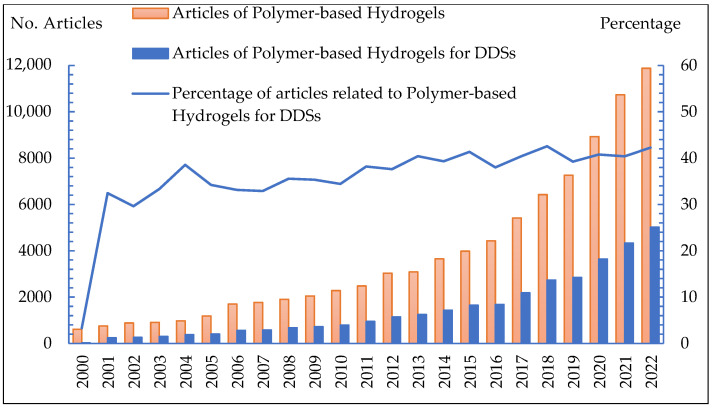
Increasing number of publications related to polymer-based hydrogels and polymer-based hydrogels for drug delivery systems from 2000 to 2022.

The increasing number of research articles on polymer-based hydrogels, particularly in the field of drug delivery, reflects the growing interest and recognition of the potential of hydrogel-based systems in biomedical applications [11,23,101,102,233]. The unique properties of hydrogels, such as their high water content, biocompatibility, and tunable mechanical properties, make them highly suitable for drug delivery applications [11,23,142,143,144,145,146]. The graph in Figure 2 illustrates the significant growth in research publications related to polymer-based hydrogels from 2000 to 2022. The number of publications has steadily increased over the years, indicating the expanding research efforts in this field. In 2005, there were less than 1000 publications per year on polymer-based hydrogel materials in general, with less than 400 publications per year specifically focused on polymer-based hydrogels for drug delivery, accounting for less than 35% of the total. Between 2005 and 2015, there was a steady increase in research publications. In 2015, the number of publications on polymer-based hydrogel materials reached nearly 4000, with around 1650 articles specifically dedicated to polymer-based hydrogels for drug delivery, accounting for approximately 38% of the total publications in the field. From 2015 to the present, there has been a remarkable jump in research related to hydrogels. For instance, in 2022, the number of publications on polymer-based hydrogel materials surpassed 12,000, with more than 5000 articles specifically focused on polymer-based hydrogels for drug delivery, accounting for over 42% of the total publications. This upward trend signifies the increasing recognition of hydrogels as a promising platform for drug delivery applications. The continuous growth in research indicates the ongoing exploration of novel hydrogel formulations, synthesis methods, drug encapsulation techniques, and delivery mechanisms. As researchers uncover new insights and address existing challenges, the field of hydrogel-based drug delivery is expected to further expand in the future, leading to the development of more advanced and efficient drug delivery systems.

Current and future research on polymer-based hydrogels applied in drug delivery systems is focused on advancing the field by addressing existing challenges and exploring innovative approaches. In the future, it is predicted that the key trends and areas of research in the polymer-based hydrogels applied for drug delivery systems will include the following:

1. Advanced drug release strategies: Researchers are developing novel drug release strategies that offer precise control over the release rate and duration of therapeutic agents. This includes the development of stimuli-responsive polymer-based hydrogels that can release drugs in response to specific triggers such as pH, temperature, enzymes, or light. Additionally, efforts are being made to incorporate drug delivery mechanisms such as sustained release, pulsatile release, and on-demand release into polymer-based hydrogel systems.
2. Targeted and site-specific delivery: Enhancing the targeting efficiency and specificity of drug delivery systems is a major focus. Researchers are exploring the incorporation of targeting ligands, such as antibodies, peptides, or aptamers, into polymer-based hydrogels to improve the delivery of drugs to specific cells, tissues, or organs. The use of external stimuli, such as magnetic fields or ultrasound, to guide the polymer-based hydrogel to the target site is also being investigated.
3. Combination therapy: Polymer-based hydrogels are being studied for their potential to deliver multiple therapeutic agents simultaneously, enabling combination therapy. This includes the co-delivery of drugs with different mechanisms of action or the incorporation of drugs and biological molecules, such as growth factors or gene therapies, within the polymer-based hydrogel matrix. Combination therapy can enhance therapeutic efficacy, reduce side effects, and overcome drug resistance.
4. Bioactive polymer-based hydrogels: Researchers are incorporating bioactive molecules, such as peptides or growth factors, into hydrogels to promote tissue regeneration and healing. These bioactive polymer-based hydrogels can provide a suitable microenvironment for cells, promote cell adhesion and proliferation, and support tissue integration. Such polymer-based hydrogels hold promise for applications in wound healing, tissue engineering, and regenerative medicine.
5. Nanotechnology and nanoparticles: Nanoparticles and nanotechnology are being integrated into polymer-based hydrogel systems to enhance drug loading, stability, and release. Nanoparticles, such as liposomes, polymeric nanoparticles, or inorganic nanoparticles, can be incorporated within the polymer-based hydrogel matrix or used as carriers to encapsulate drugs. These nanoparticles can provide controlled release profiles, protect drugs from degradation, and enable targeted delivery.
6. 3D printing and additive manufacturing: Advances in 3D printing and additive manufacturing techniques have opened new avenues for the fabrication of complex and customized hydrogel-based drug delivery systems. Researchers are exploring the use of 3D printing to create patient-specific implants, scaffolds, or drug-loaded structures with precise control over shape, architecture, and drug distribution.
7. Smart polymer-based hydrogels and sensors: The integration of smart polymer-based hydrogels with sensing capabilities is an emerging area of research. These hydrogels can detect and respond to specific biological or environmental cues, such as pH changes, enzyme activity, or the presence of specific biomarkers. By incorporating sensors into polymer-based hydrogels, the real-time monitoring of drug release, therapeutic efficacy, or disease progression can be achieved.
8. Biodegradability and sustainability: Researchers are focusing on developing biodegradable hydrogels that can degrade in a controlled manner, eliminating the need for surgical removal. The use of biocompatible and sustainable materials, such as natural polymers or biomimetic materials, is also gaining attention to reduce the environmental impact of hydrogel-based drug delivery systems.

The future of research into polymer-based hydrogels for drug delivery systems lies in designing more sophisticated, targeted, and patient-specific approaches. The integration of advanced technologies, materials, and knowledge from various disciplines will continue to drive innovation in this field, leading to improved therapeutic outcomes and personalized medicine.

## 4. Conclusions

Polymer-based hydrogels have emerged as a promising class of materials for drug delivery systems, offering unique properties that make them highly suitable for biomedical applications. Their high water content, soft and elastic texture, and biocompatibility make them well suited for interacting with biological systems. Throughout this review, we have explored various hydrogel-based drug delivery systems and highlighted their advantages. One key advantage is ability of hydrogels to achieve sustained drug release, ensuring a controlled and prolonged delivery of therapeutic agents. This is particularly beneficial for drugs that require a specific release profile or long-term treatment. Another advantage is the potential for targeted drug delivery. By incorporating targeting moieties or modifying the polymer-based hydrogel’s properties, drugs can be delivered specifically to a desired site within the body, minimizing systemic exposure and reduced toxicity. Moreover, the increasing number of publications on polymer-based hydrogels in the past two decades indicates the growing interest and importance of these materials in the biomedical field. This active research and development contributes to the continuous improvement of polymer-based hydrogel properties and functionalities. It is expected that further advancements will lead to new and innovative applications of polymer-based hydrogels in the future. Beyond drug delivery, polymer-based hydrogels have shown potential in tissue engineering, wound healing, and biosensing applications. Their biocompatibility and ability to mimic the extracellular matrix make them valuable scaffolds for tissue regeneration and repair. Additionally, polymer-based hydrogels can be designed to respond to specific biological signals, enabling their use in biosensors for the detection and monitoring of biomarkers. Polymer-based hydrogels for drug delivery systems hold great promise in revolutionizing the treatment of various diseases and medical conditions. By improving drug efficacy, reducing side effects, and enhancing patient compliance, polymer-based hydrogels have the potential to significantly improve healthcare outcomes. Additionally, their versatility and wide range of applications make them an exciting area of research and development with promising opportunities to advance medical science.

## Figures and Tables

**Table 1:** Classification of drug delivery systems [3,8,11,112,113].

Types of Drug Delivery Systems	Categories
Conventional or traditional drug delivery systems	Oral delivery
	Buccal or sublingual delivery
	Rectal delivery
	Intravenous delivery
	Subcutaneous delivery
	Intramuscular delivery
Novel or controlled-release drug delivery systems	Rate-preprogrammed
	Activation-modulated
	Feedback-regulated
	Site-targeting

**Table 2:** The advantages and disadvantages of traditional drug delivery methods [3,8,11,112,113].

Traditional Drug Delivery Methods	Advantages	Disadvantages
Oral delivery	Convenience in administration Non-invasive Accurate and measured dose Unit dosage form Higher compliance Cheap for the patient	Unconscious patients cannot take a dose Low permeability Degradation by gastro-intestinal enzymes First pass metabolism Irregular absorption
Buccal or sublingual delivery	Bypass first pass metabolism Rapid absorption Low enzymatic activity	Discomfort during dissolution Probability of swallowing—loss of effect Small doses
Rectal delivery	Bypass first pass metabolism Useful for kids and children	Absorption depends on disease state Degradation by bacterial flora Uncomfortable
Intravenous delivery	Drug 100% bioavailable Rapid response Can administer drugs degradable in stomach By-passes first pass metabolism	Invasive Trained personnel Possible toxicity due to incorrect dosing Sterility
Subcutaneous delivery	Patient self-administration Slow complete absorption Bypass first pass metabolism when placed at lower part of rectum	Invasive Irritation Inflammation Maximum dose volume—2 mL
Intramuscular delivery	Drug is absorbed slowly, so prolonged effect Larger volume than subcutaneous Bypass first pass metabolism	Invasive—patient discomfort Irritation Inflammation May require some training

**Table 3:** Classifications of polymer-based hydrogels in different ways.

No.	Classifications of Polymer-Based Hydrogels	Type of Polymer-Based Hydrogels
01	Classification of polymer-based hydrogels based on origin data	Natural polymer-based hydrogels
		Synthetic polymer-based hydrogels
		Hybrid polymer-based hydrogels
02	Classification of polymer-based hydrogels based on composition	Homopolymer polymer-based hydrogels
		Copolymer polymer-based hydrogels
		Multipolymer polymer-based hydrogels
		Interpenetrating network (IPN) polymer-based hydrogels
03	Classification of polymer-based hydrogels based on ionic charge	Neutral polymer-based hydrogels
		Ionic polymer-based hydrogels
		Ampholytic polymer-based hydrogels
04	Classification of polymer-based hydrogels based on pore size	Macroporous polymer-based hydrogels
		Mesoporous polymer-based hydrogels
		Microporous polymer-based hydrogels
05	Classification of polymer-based hydrogels based on physical appearance	Matrix polymer-based hydrogels
		Film polymer-based hydrogels
		Microsphere polymer-based hydrogels
		Nanoparticle polymer-based hydrogels
		Polymer-based hydrogel beads
06	Classification of polymer-based hydrogels based on crystallinity	Amorphous polymer-based hydrogels
		Semi-crystalline polymer-based hydrogels
07	Classification of polymer-based hydrogels based on crosslinking	Chemical crosslinking polymer-based hydrogels
		Physical crosslinking polymer-based hydrogels
08	Classification of polymer-based hydrogels based on external stimuli response	Temperature-responsive polymer-based hydrogels
		pH-responsive polymer-based hydrogels
		Light-responsive polymer-based hydrogels
		Electric-field-responsive polymer-based hydrogels
		Magnetic-field-responsive polymer-based hydrogels

## Data Availability

Data are contained within the article.
